# The use of cold atmospheric plasma/J-Plasma in the management of rhinophyma: A case report

**DOI:** 10.1016/j.jpra.2025.02.010

**Published:** 2025-02-22

**Authors:** Abdullahi A. Adan, Najma Jelle

**Affiliations:** a Plastic Aesthetic and Reconstructive Surgeon, Platinum Surgery Centre, Department of Surgery, University of Nairobi, P.O. Box 76239-00508, Nairobi, Kenya; b MBChB, Department of Surgery, University of Nairobi, P.O. Box, 30197-00100, Nairobi, Kenya

**Keywords:** Rhinophyma, Cold atmospheric plasma, J-Plasma

## Abstract

Rhinophyma is a disfiguring swelling on the nose occurring due to the proliferation of sebaceous glands. The purpose of this case report is to describe clinical findings in a patient who presented with rhinophyma in the Kenyan setting and the application of cold atmospheric plasma/J-Plasma technology in management. Aesthetic outcome is an important consideration when choosing between surgical management options and excision using J-Plasma is associated with desirable aesthetic outcome.

## Introduction

### Background

Rhinophyma is a tumor-like swelling on the nose that occurs as a result of proliferation of sebaceous glands and underlying connective tissue.^1^ It is a benign but disfiguring condition associated with psychological distress and if severe, respiratory distress. Rosacea is the precursor condition of rhinophyma and while its pathogenesis is not well elucidated; it is caused by inflammatory reaction as a result of dysregulation of the neurovascular mechanism and innate immune responses.^1^ Rosacea can occur anywhere in the face but phymatous changes of rosacea primarily affect the nose and is more prevalent in men and fair skin populations.^2^

### Management options

Management options for mild symptoms of rosacea include topical antibiotics such as metronidazole that lessens the inflammation or oral isotretinoin that lowers sebum production and the size of the sebaceous glands.^1^ For advanced features of Rosacea including rhinophyma surgical treatment is inevitable and involves resection of the hypertrophic tissue with a wide range of surgical options available. These options include Excision with a blade, cryosurgery, surgical ablation as well as laser surgery.^3^ In addition to these options is the new cold atmospheric plasma technology reported to have been used for rhinophyma treatment in a few cases with successful outcome.^4^^,^^5^

The proposed benefit of using J-Plasma in treatment of rhinophyma is that it allows for thermal precision reducing collateral damage to healthy tissue during excision.^4^ The system allows the surgeon to ablate tissue layer by layer. Furthermore, plasma energy generates an antimicrobial effect thus reducing the risk of infection. All these features coupled together help in achieving desirable aesthetic outcome post excision, reduce the risk of scarring and allows faster healing.

## Case presentation

71-year-old, Kenyan-Australian male presented with history of swelling on the nose for three years that was of gradual onset with associated difficulty in breathing low self-esteem and feeling dissatisfied with his appearance. He had a 10-pack year history of smoking and history of hip replacement and spine surgery but no known comorbidities, no family history of similar condition, and no history of alcohol use.

**Figure 1:**
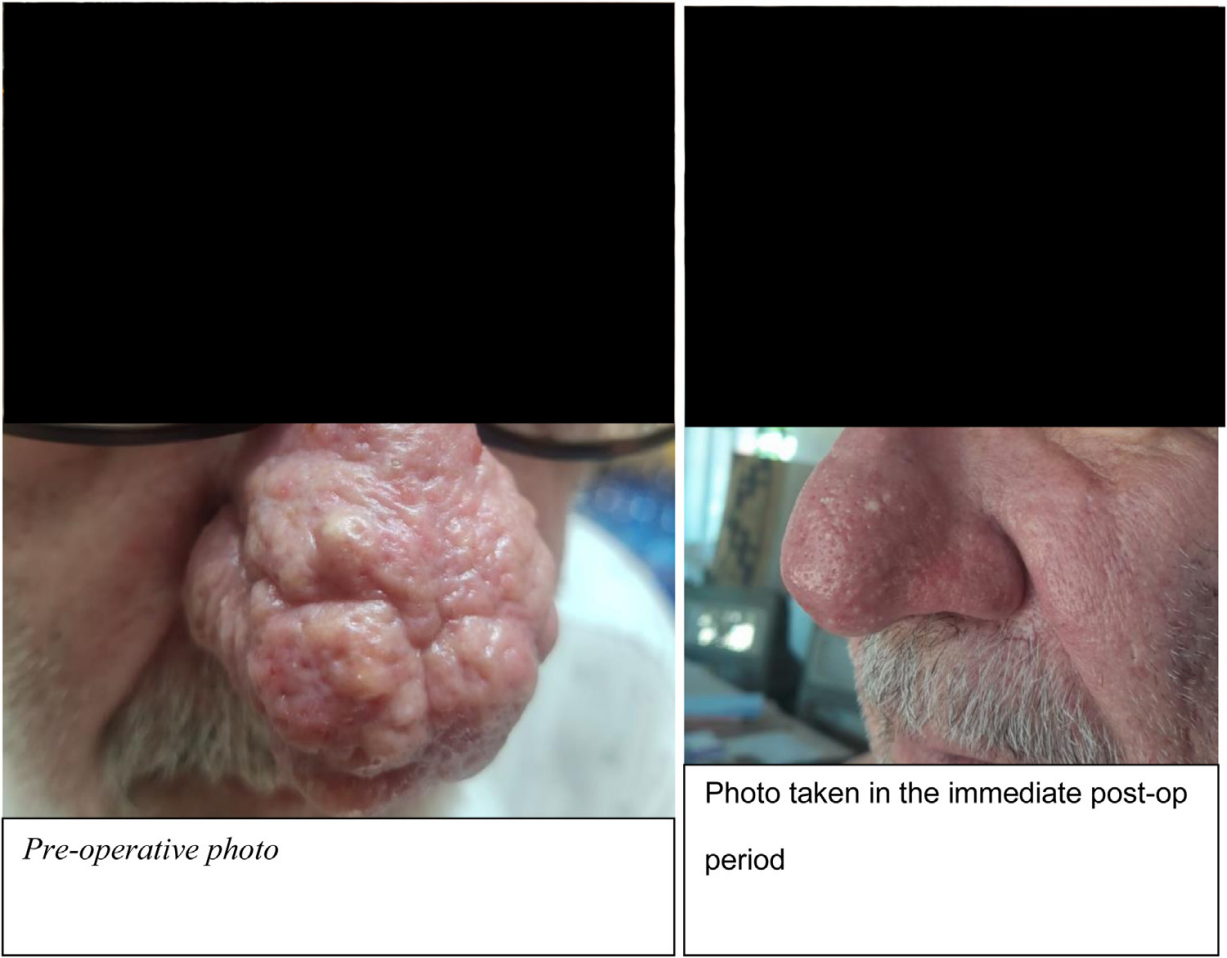
Right side of photo showing examination findings of rhinophyma and left side showing findings after excision with J-Plasma.

Examination revealed enlargement of the nose with firm, lobular, rubbery, bulbous and non-tender growth extending from the dorsum of the nose to the tip and bilateral nasal alae. The skin overlying the nose was coarse with enlarged pores and areas with pustules as seen in (Figure 1). General and systemic examination was normal. Diagnosis of rhinophyma was made clinically and confirmed by histology which revealed sebaceous gland hyperplasia. Complete Blood Count, Liver and kidney function test as well as cardiac evaluation done were normal.

### Intervention

After extensive consultation and literature review, a decision to use J-Plasma in the management of this patient was reached. The J-Plasma device used in this case from Bovie Medica Co-operation comprises of a hand piece that generates plasma energy through ionization of helium gas. The patient was put under general anesthesia, the skin prepared, cleaned and a sterile field established. Standard Personal protective measures including face mask and eye shield to protect the eye from potential plasma energy exposure were used by the surgical team but no further protective gear was needed.

The J-Plasma device was set at a power of 65 % initially and subsequently 70 %, then 75 % towards the end of the procedure. All the excessive tissue of hypertrophied sebaceous glands were excised up to the level of normal tissue. This was done by the surgeon holding the handpiece just above, approximately 4 mm from the hypertrophied tissue and applying a continuous sweeping motion of focused plasma energy in each pass to ensure even ablation while minimizing the risk of thermal damage. A total of three passes were sufficient to remove the hypertrophied tissue with a controlled depth of ablation approximately 0.5–1 mm in each pass followed by wiping off the ablated tissue using a sterile gauze after each pass to monitor the progress. During ablation of the hypertrophied tissue, the nasal surface was sculpted to achieve the normal contour of the nose. Hemostasis was instantaneous due to the coagulative ability of plasma energy. The Surgical site was left raw to granulate with hydrocolloid gel (Intracite gel- Smith and Nephew) applied to keep the wound environment moist thereby enhancing healing and epithelization. This was followed by dressing using Bactigras (also from Smith and Nephew), a paraffin dressing with chlorhexidine to offer protection while retaining moisture. Follow-up review at 1 week, 2 weeks and 1 month revealed no complications. The patient was followed up every 3 months for the first year after surgery and twice in the subsequent year with no recurrence of the rhinophyma noted.

J-Plasma technology has significant benefits over traditional ablative and excisional surgical techniques due to the utility of ablative function, coagulative property and the ability to enhance subdermal contraction to aid in sculpting all in one device. This, coupled with precise control over the depth of ablation and minimized risk of thermal injury due to the low temperature ablation provides the benefit of better cosmetic outcomes. In Comparison to CO2 Laser ablation, J-Plasma treatment offers precise ablation while also reducing the risk of collateral thermal injury. While it's use in our case was facilitated by availability and promising technical abilities as documented in literature, we believe that J-Plasma has the potential to become the routine treatment option of rhinophyma. However, Further research with a larger study population and longer follow-up time is needed to determine its use as the standard management option in rhinophyma and its benefit over other surgical techniques.

## Discussion

In the management of facial tumors, it is important to choose a management option that ensures the best aesthetic outcome for the patient and among these options is the use of J-Plasma.^4^ Excision with J-Plasma preserves the underlying healthy tissue by reducing the risk of thermal injury due to the low thermal energy released by plasma and thermal precision level achieved during surgery. Furthermore, plasma energy contains antimicrobial properties mediated by reactive oxygen species and has been used widely to promote wound healing in surgery. This added benefit reduces the risk of post-operative infection and allows for faster wound healing. There have been very few cases reported that used J-Plasma in managing rhinophyma and all reported good results with excellent patient satisfaction.^4^^,^^5^ In Our case, the immediate post op results after excision of the growth were satisfactory and the patient was happy with his appearance and no complications noted.

## Conclusion

The use of cold atmospheric plasma in the management of rhinophyma offers satisfactory post-operative results and cosmetic outcome.

## Ethical consideration

Consent was obtained from the patient to publish the findings in this case. The details of the case including the images have been anonymized.

## Funding

None.

## Conflict of interest

None declared.
